# Optical Diffraction in Close Proximity to Plane Apertures. I. Boundary-Value Solutions for Circular Apertures and Slits

**DOI:** 10.6028/jres.107.028

**Published:** 2002-08-01

**Authors:** Klaus D. Mielenz

**Affiliations:** National Institute of Standards and Technology, Gaithersburg, MD 20899-8440

**Keywords:** boundary value theory, circular apertures, diffraction, Kirchhoff, near zone, optics, polarization, Rayleigh, Sommerfeld, scalar wave functions, slits

## Abstract

In this paper the classical Rayleigh-Sommerfeld and Kirchhoff boundary-value diffraction integrals are solved in closed form for circular apertures and slits illuminated by normally incident plane waves. The mathematical expressions obtained involve no simplifying approximations and are free of singularities, except in the aperture plane itself. Their use for numerical computations was straightforward and provided new insight into the nature of diffraction in the near zone where the Fresnel approximation does not apply. The Rayleigh-Sommerfeld integrals were found to be very similar to each other, so that polarization effects appear to be negligibly small. On the other hand, they differ substantially at sub-wavelength differences from the aperture plane and do not correctly describe the diffracted field as an analytical continuation of the incident geometrical field.

## 1. Introduction

Diffraction problems in optics typically involve distances from the diffracting screen which are large in comparison to the wavelength of light. Accordingly the Fresnel and Fraunhofer approximations of the principal classical diffraction integrals are well documented, but so far no workable expressions have been available for computations in the near zone. The aim of the present paper is to develop mathematical procedures for the latter purpose and use them to study the behavior of these integrals in the proximity of plane apertures.

We begin by citing the classical scalar expressions for analyzing optical diffraction by an aperture; namely, Kirchhoff’s integral equation [1]

$$U\left(P\right)=-\frac{1}{4\pi }\int_{S}\text{dQ}[U\left(Q\right)\frac{\partial }{\partial n}\left(\frac{e^{ikQP}}{QP}\right)-\frac{\partial U\left(Q\right)}{\partial n}\frac{e^{ikQP}}{QP}]$$ (1)

and, alternatively, the Rayleigh-Sommerfeld integral equations [2, 3, 4]

$$\begin{array}{l} U\left(P\right)=-\frac{1}{2\pi }\int_{S}\text{dQ}\frac{\partial U\left(Q\right)}{\partial n}\frac{e^{ikQP}}{QP}\\ =\frac{1}{2\pi }\int_{S}\text{dQ}\;U\left(Q\right)\frac{\partial }{\partial n}\left(\frac{e^{ikQP}}{QP}\right), \end{array}$$ (2)

**Fig. 1:**
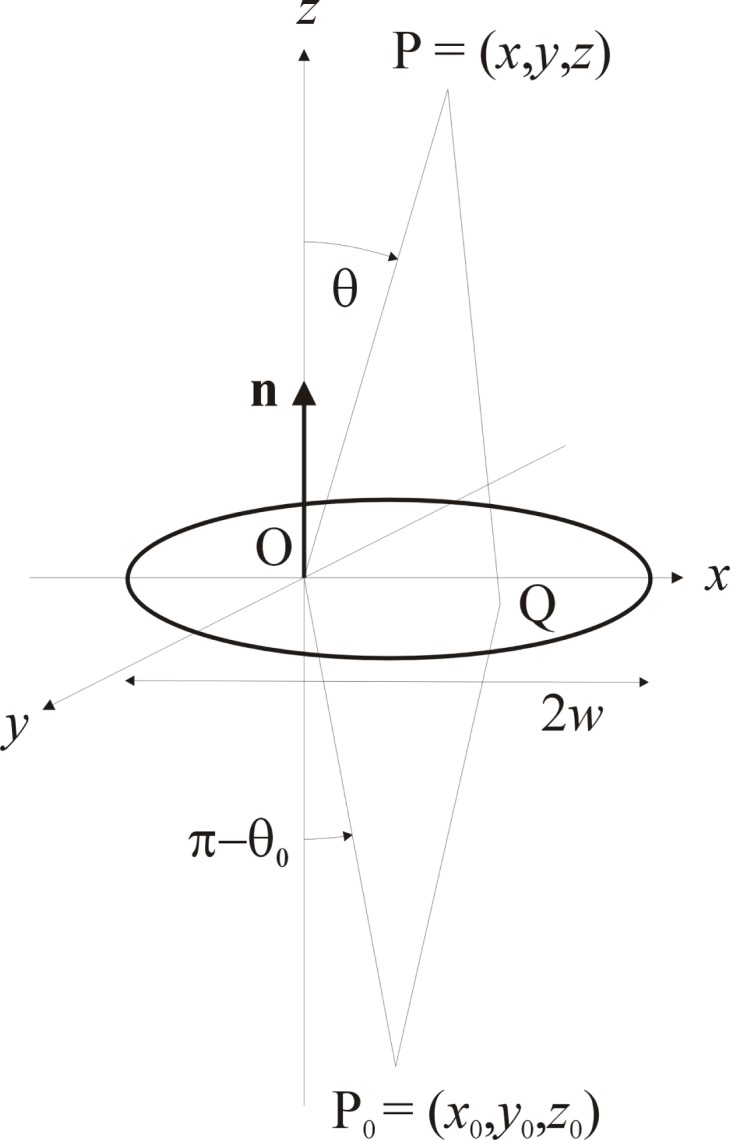
Basic geometry for plane aperture of width 2*w*. ***n*** = aperture normal, O = coordinate origin, P_0_ = point source, P = point of observation.

In these equations, *U* is a scalar wave function, S is a closed surface containing a plane aperture A located in the *xy*-plane of a cartesian coordinate system as indicated in Fig. 1, P = (*x*, *y*, *z*) is the point of observation (*z ≥* 0), Q = (*ξ*, ***η***, 0) is a point on A, *QP* is the distance between them, ***n*** is the aperture normal pointing in the direction of the positive *z*-axis, and *k* = 2π/λ is the circular wavenumber of monochromatic light with wavelength λ.

## 2. Background

The Kirchhoff and Rayleigh-Sommerfeld integral equations (1) and (2) are alternative forms of the theorem of Helmholtz [5], which expresses Huygens’ principle in terms of a scalar wave function *U* and its normal derivatives without assuming specific attributes of this function, except that it is continuous and twice differentiable with continuous derivatives and obeys the homogeneous wave equation,

$$\Delta U+k^{2}U=0,$$ (3)

on and within the closed surface S. As Helmholtz’ theorem by itself is insufficient to provide a unique solution, it is necessary to impose additional constraints on *U* by prescribing its boundary values on S.

Kirchhoff considered a “*black screen which neither reflects nor transmits light*.” He assumed, plausibly, that in this case the incident field vanishes altogether on the opaque portion of the screen and is equal to the unperturbed incident field *U*_geom_(Q) inside the aperture. Thus,

$$U\left(Q\right)=0\;\text{and}\;\frac{\partial U\left(Q\right)}{\partial n}=0,\;\text{when}\;Q\;\notin \;A,$$ (4a)

$$U\left(Q\right)=U_{\text{geom}}\left(Q\right),\;\text{when}\;Q\;\in A,$$ (4b)

so that Eq. (1) is reduced to Kirchhoff’s familiar formula, where the integration extends over the aperture area A only, and *U* is replaced by *U*_geom_ in the integrand. As it turned out, Kirchhoff’s solution is mathematically flawed. Poincaré [6] discovered that it contradicts itself and predicted that it will not reproduce the assumed boundary conditions Eq. (4a, b).

Sommerfeld [4, 7] recognized that these difficulties are due to the fact that *U* and ∂*U*/∂***n*** cannot both vanish on any finite portion of the closed surface S unless *U* is everywhere identically equal to zero. He remedied the problem by deriving the integral equations [Eq. (2)] which require only the boundary values of either *U* or ∂*U*/∂***n*** to specify a solution. Thus he assumed, instead of Eq. (4a),

$$\frac{\partial U\left(Q\right)}{\partial n}=0\;\text{or}\;U\left(Q\right)=0,\;\text{when}\;Q\;\notin A,$$ (5a)

$$U\left(Q\right)=U_{\text{geom}}\left(Q\right),\;\text{when}\;Q\in \;A,$$ (5b)

and applied these conditions separately to the first and second Eqs. (2) Except for a passing reference to “*shiny screens*,” Sommerfeld did not explicitly associate his theory with the diffraction of polarized light but merely offered the solution appearing in Eq. (5e), below, as a mathematically improved alternative to Kirchhoff’s formula. On the other hand, Rayleigh [2,3] emphasized that the screen must be supposed to be a perfect metallic reflector so that the boundary conditions Eq. (5a) pertain to *p*- and *s*-polarized incident light, respectively. He emphasized, further, that these conditions apply on the dark side of the screen only, while on the lit side it must be assumed that

$$U_{\text{geom}}^{\left(p\right)}\left(Q\right)=0\;\text{or}\;\frac{\partial U_{\text{geom}}^{\left(s\right)}\left(Q\right)}{\partial n}=0,\;\text{when}\;Q\;\notin A.$$ (5c)

According to Maxwell’s equations, *U*^(^*^p^*^)^ and ∂*U*^(^*^s^*^)^/∂***n*** must be continuous at the screen and hence it follows that, taken together, the conditions of Eqs. (5a) and (5c) stipulate that for either state of polarization *U* and ∂*U*/∂***n*** must both zero on the dark side of the screen. This is the same as Kirchhoff’s boundary condition of Eq. (4a), but without mathematical contradictions. The final result for the Rayleigh-Sommerfeld integrals is

$$U_{\text{RS}}^{\left(p\right)}\left(P\right)=-\frac{1}{2\pi }\int_{A}\text{dQ}\frac{\partial U_{\text{geom}}\left(Q\right)}{\partial n}\frac{e^{ikQP}}{QP},$$ (5d)

$$U_{\text{RS}}^{\left(s\right)}\left(P\right)=\frac{1}{2\pi }\int_{S}\text{dQ}\;U_{\text{geom}}\left(Q\right)\frac{\partial }{\partial n}\left(\frac{e^{ikQP}}{QP}\right),$$ (5e)

where, as in Kirchhoff’s theory, the screen itself does not contribute to the integrals. According to an analysis performed by Mukunda [8], these expressions do recover the assumed boundary conditions in Eqs. (5a–c) as P → Q, in the sense that $U_{\text{RS}}^{\left(s\right)}$ RS replicates the assumed value of *U*_geom_ but not necessarily the compatible value of ∂*U*_geom_/∂***n***, and the converse is true for $U_{\text{RS}}^{\left(p\right)}$.

It should also be noted that Kirchhoff’s solution is simply the arithmetic mean of the Rayleigh-Sommerfeld solutions,

$$u_{K}\left(P\right)=\frac{1}{2}[u_{\text{RS}}^{\left(p\right)}(P)+u_{\text{RS}}^{\left(s\right)}(P)],$$ (6a)

and that in the so-called Fresnel limit they are all reduced to one and the same expression. For a point source P_0_ = (*x*_0_, *y*_0_, *z*_0_), as shown in Fig. 1, and assuming that the distances −*z*_0_ and *z* are large in comparison to the aperture width 2*w* and the wavelength λ, and that cos(π − *θ*_0_) and cos θ are essentially equal to 1, one finds [8]

$$U_{K}\left(P\right)\approx U_{\text{RS}}^{\left(p\right)}\left(P\right)\approx U_{\text{RS}}^{\left(s\right)}\left(P\right)\approx U_{F}\left(P\right)=\frac{i\sqrt{I_{0}}e^{ik\left(z-z_{0}\right)}}{zz_{0}}{\int_{A}\text{dQe}}^{ik\Delta \left(Q\right)},$$ (6b)

where *I*_0_ is the radiant intensity of the source and Δ(Q) is a second-order approximation of the path difference (*P*_0_*Q* + *QP*) − (*P*_0_*O − OP*). It may be estimated that this approximation is accurate to 1 % or better when *z* > 20*w* and *z* > 20λ so that it is usually satisfied for narrow apertures and short wavelengths; say, 2*w* = 0.1 mm and λ= 500 nm, as used for pinhole imagery or classroom experiments. In these cases the Rayleigh-Sommerfeld and Kirchhoff solutions will hardly be needed in their rigorous forms, but on the other hand the reliability of the Fresnel approximation is doubtful for large apertures and wavelengths. It may not be applicable in the focal planes of fast lenses or in the case of large apertures used in radiometry and photometry, especially in the infrared and microwave regions.

**Fig. 2:**
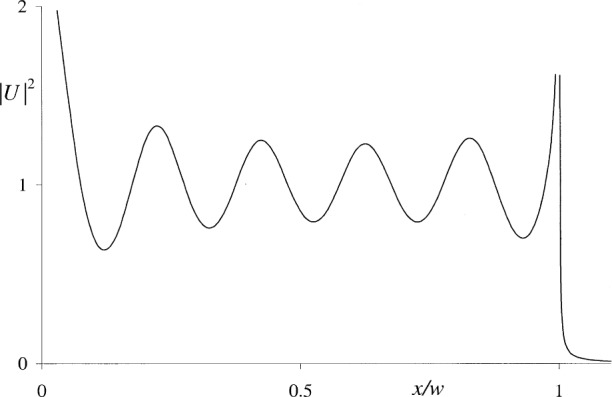
Approximate behavior of Kirchhoff’s integral *U*_K_ inside a circular aperture of diameter 2*w* = 10*λ*. Computed from Eq. (A16) of Ref. [10].

**Fig. 3:**
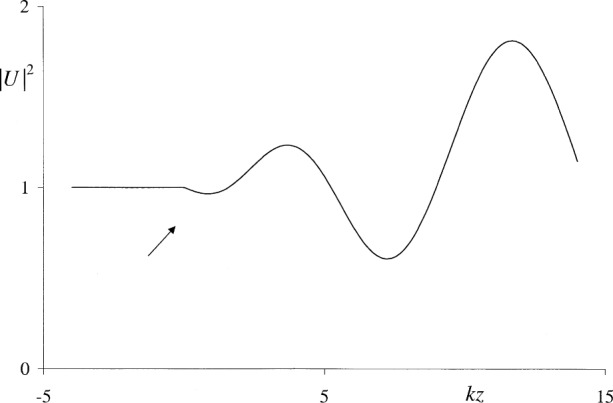
Behavior of the Rayleigh-Sommerfeld integral $u_{\text{RS}}^{\left(s\right)}$ for axial points near a circular aperture of diameter 2*w* = 10*λ*. Computed from Eq. (7) of Ref. [12].

Apart from the above, it appears that the behavior of the Kirchhoff and Rayleigh-Sommerfeld integrals in the aperture plane has not been documented in the literature except in two isolated cases. Wolf and Marchand [10] derived a closed expression for Kirchhoff’s integral *U*_K_ inside a circular aperture illuminated by a normally incident plane wave, using the Maggi-Rubinowicz transformation [11] of *U*_K_ and a stationary-phase approximation. As shown in Fig. 2, this expression gives a fair indication of an oscillatory behavior of *U*_K_ in the aperture plane but exhibits spurious singularities at the aperture center and rim. Osterberg and Smith [12] found a closed expression for $U_{\text{RS}}^{\left(s\right)}$ at axial points of observation behind a circular aperture and confirmed that it does represent a continuous extension of the incident field into the half space *z* > 0. On the other hand, it is evident from Fig. 3 that the combined field is not continuously differentiable at *z* = 0, showing that $U_{\text{RS}}^{\left(s\right)}$ still does not fully meet the requirements of Helmholtz’ theorem. There appear to be no known solutions for $U_{\text{RS}}^{\left(p\right)}$, but based on Mukunda’s work it is clear that $U_{\text{RS}}^{\left(p\right)}=2U_{K}-U_{\text{RS}}^{\left(s\right)}$ is discontinuous in the aperture plane because *U*_K_ is discontinuous.

In addition to these publications, the pertinent literature also contains a considerable number of papers that were, for the most part, intended to “save” Kirchhoff’s theory in one way or another. For example, Kottler [13] regarded Kirchhoff’s integral as the rigorous solution of a “saltus” problem in which the wave function *U* has prescribed discontinuities rather than boundary values at the aperture screen.^1^ Kottler’s theory involved the above-mentioned Maggi-Rubinowicz transformation, and subsequently the singularities inherent in the latter led to the revival of a belief that diffraction can be attributed to “boundary diffraction waves” emerging from the edges of aperture screens. Marchand and Wolf [14, 15] asserted that the inconsistencies of Kirchhoff’s integral are only apparent and developed a theory in which *U*_K_ is expressed in terms of vector potentials that have singularities even in free space. Born [16] suggested the possibility that Kirchhoff’s formula may be only one in a series of successive approximations, and Franz [17] re-derived it by an iterative method in which the discontinuities of previous solutions are regarded as secondary sources of light. All in all, this curious exchange of conjectures has raised more questions than it has answered. Most certainly, it has not addressed the concerns of laboratory physicists in search of a “best” theory for practical application.

## 3. Mathematical Expressions and Numerical Results

### 3.1 General

In order to analyze the behavior of the Kirchhoff and Rayleigh-Sommerfeld integrals in the proximity of apertures it is necessary to derive usable expressions for computations in the near zone. For this purpose and to keep the calculations simple, it will be assumed in the following that the incident field is a normally incident plane wave so that the geometrical field in the aperture is given by

$$U_{\text{geom}}\left(Q\right)=\sqrt{E_{0}},\frac{\partial U_{\text{geom}}\left(Q\right)}{\partial n}=ik\sqrt{E_{0}},$$ (7a)

where *E*_0_ denotes irradiance, and Eqs. (5d) and (5e) can be written in normalized form as

$$u_{\text{RS}}^{\left(p\right)}\left(P\right)\equiv U_{\text{RS}}^{\left(p\right)}\left(P\right)/\sqrt{E_{0}}=-\frac{ik}{2\pi }\int_{A}\text{dQ}\frac{e^{ikQP}}{QP},$$ (7b)

$$u_{\text{RS}}^{\left(s\right)}\left(P\right)\equiv U_{\text{RS}}^{\left(s\right)}\left(P\right)/\sqrt{E_{0}}=\frac{1}{2\pi }\int_{A}\text{dQ}\frac{\partial }{\partial n}\left(\frac{e^{ikQP}}{QP}\right)=\frac{1}{ik}\frac{\partial u_{\text{RS}}^{\left(p\right)}}{\partial z}.$$ (7c)

The simplicity of these relationships is a fortunate consequence of having assumed a normally incident plane wave. Once the above expression for $u_{\text{RS}}^{\left(p\right)}$ has been evaluated, the solution for $u_{\text{RS}}^{\left(s\right)}$ follows by differentiation with respect to *z*, and then Kirchhoff’s integral [Eq. (1)] is co-determined as the arithmetic mean defined in Eq. (6a). There is little doubt that other forms of the incident field would have led to considerably more complicated expressions without adding to the physical significance of the results. In the following, Eqs. (7b) and (7c) will be reduced to single integrals for the respective cases of circular apertures and slits.

### 3.2 Circular Aperture

**Fig. 4:**
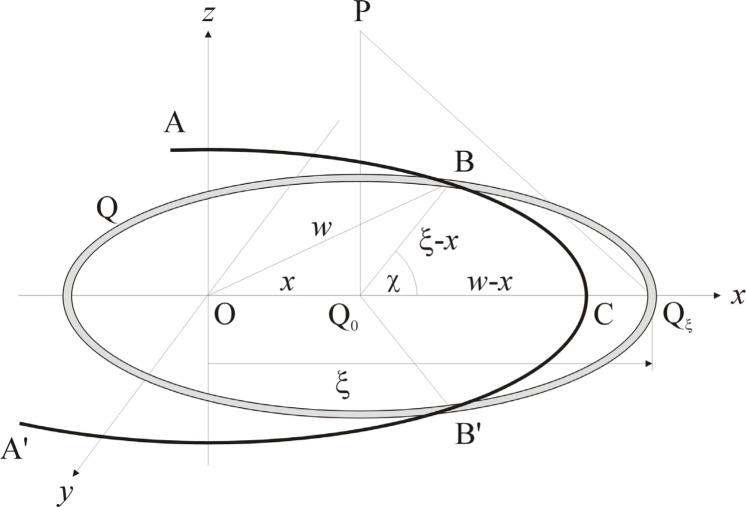
Geometrical notation used for circular apertures.

Let ABCB′A′ be the rim of a circular aperture of radius 2*w* which is centered on the origin O of a cartesian coordinate system, as shown in Fig. 4. As the corresponding diffraction pattern must be rotationally symmetrical about the *z*-axis, it will be sufficient to consider its variation in the *xz*-plane and the point of observation may be chosen as *P* = (*x*, 0, *z*). The integrals [Eqs. (7b) and (7c)] may then be reduced to single integrals by defining the area elements dQ so that they are concentric with the projection Q_0_ = (*x*, 0, 0) of P onto the aperture plane and coincide with the circles QBQ*_ξ_*B′ shown in the figure, where Q*_ξ_*= (*ξ*, 0, 0) is the right-most point at which these circles intersect the *x*-axis. Accordingly, the phases *kQP* will be constant and equal to

$$\beta \equiv kQP=kQ_{\xi }P=k\sqrt{{\left(\xi -x\right)}^{2}+z^{2}}$$ (8a)

everywhere on these area elements and the integration can be carried out over the points *Q_ξ_* alone. As also indicated in Fig. 4, these area elements are in general not fully contained in the aperture and must therefore be evaluated as

dQ=2πd(ξ−x)(ξ−x)(1−χ/π), (8b)

where 2*χ* is the angle subtended by the obstructed arc BQ*_ξ_*B′ and is given by

$$\cos\chi =\frac{w^{2}-x^{2}-{\left(\xi -x\right)}^{2}}{2x\left(\xi -x\right)},$$ (8c)

or *χ*= 0 or π, as appropriate, when the right-hand side of Eq. (8c) exceeds ±1. Consequently, the integrals [Eqs. (7b) and (7c)] can be expressed in the form

$$u_{\text{RS}}^{\left(p\right)}\left(x,z\right)=-ik^{2}\int d\left(\xi -x\right)\left(\xi -x\right)\left(1-\chi /\pi \right)\frac{e^{i\beta }}{\beta ,}$$ (8d)

$$u_{\text{RS}}^{\left(s\right)}\left(x,z\right)=\frac{1}{ik}\frac{\partial u_{\text{RS}}^{\left(p\right)}}{\partial z}=k^{2}z\int d\left(\xi -x\right)\left(\xi -x\right)\left(1-\chi /\pi \right)\left(\frac{1}{\beta }-i\right)\frac{e^{i\beta }}{\beta^{2}}$$ (8e)

the limits of integration being from 0 to *w* + *x* when *x ≤ w* and from *x − w* to *x* + *w* when *x ≥ w*.

For the special case of axial points of observation (*x* = 0) the angle χ defined by Eq. (8c) is zero, so that Eq. (8d) can be solved in closed form. On substitution of i*β* as a new integration variable and subsequent differentiation with respect to *z*, one finds

$$u_{\text{RS}}^{\left(p\right)}\left(0,z\right)=e^{ikz}-e^{ikW},\;W=\sqrt{w^{2}+z^{2},}$$ (9a)

$$u_{\text{RS}}^{\left(s\right)}\left(0,z\right)=e^{ikz}-\frac{ze^{ikW}}{W},$$ (9b)

the latter being identical to the above-mentioned expression derived by Osterberg and Smith [13].

**Fig. 5:**
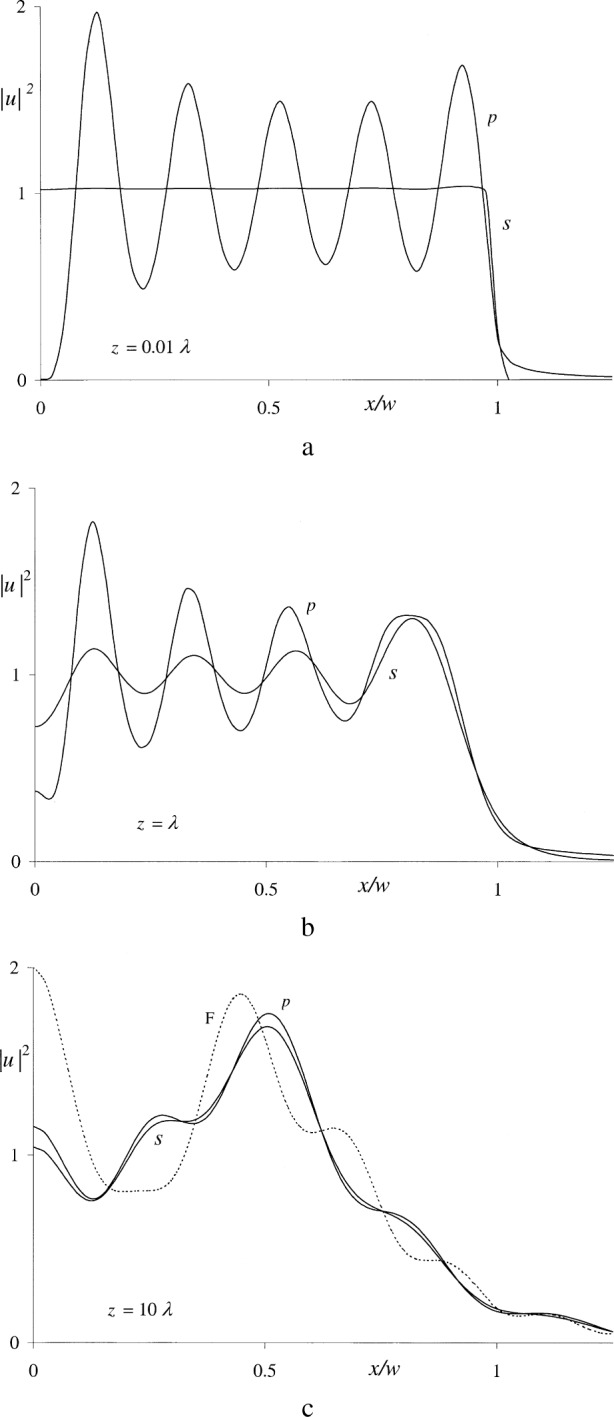
Diffraction profiles for a circular aperture of diameter 2*w* = 10λ at distances *z* ranging from 0.01λ to 10λ. The profiles shown in each graph are the squared magnitudes of the Rayleigh-Sommerfeld solutions $u_{\text{RS}}^{\left(p\right)}$ and $u_{\text{RS}}^{\left(s\right)}$ (labeled *p* and *s*), and in addition the dotted line in Fig. 5c represents the Fresnel approximation *u*_F_ (labeled F).

For *x ≠* 0, the numerical integration methods described in Ref. [18] were used to find the real and imaginary parts of Eqs. (8d) and (8e) for a small circular aperture of diameter 2*w* = 10λ at the distances *z* = 0.01λ, λ, and 10λ. The results obtained are shown in Figs. 5a though 5c and will be discussed in Sec. 4.

### 3.3 Slit

**Fig. 6:**
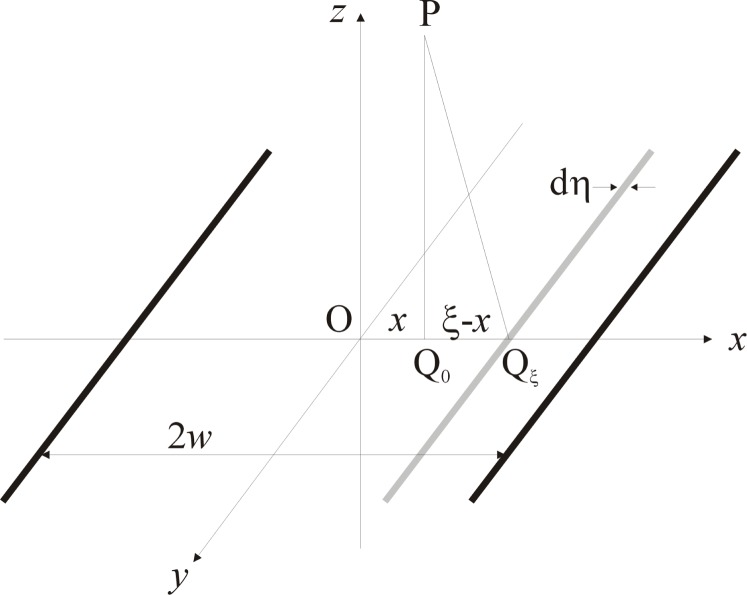
Geometrical notation used for slits.

Next we consider a diffracting slit of width 2*w*, centered in the *xy*-plane of a rectangular coordinate system as indicated in Fig. 6. The corresponding diffraction pattern will consist of straight bands which are parallel to the slit jaws, and thus it will again be sufficient to compute its variation in the *xz*-plane. For a given point of observation *P* = (*x*, 0, *z*) and for arbitrary aperture points *Q* = (*ξ*,*η*, 0), Eq. (7b) becomes

$$u_{\text{RS}}^{\left(p\right)}\left(x,z\right)=-\frac{ik}{2\pi }\int_{-w}^{w}d\xi \int_{-\infty }^{\infty }d\left(k\eta \right)\frac{e^{ik\sqrt{{\left(\xi -x\right)}^{2}+\eta^{2}+z^{2}}}}{k\sqrt{{\left(\xi -x\right)}^{2}+\eta^{2}+z^{2}}}=\frac{k}{2}\int_{-w-x}^{w-x}d\left(\xi -x\right)H_{0}^{\left(1\right)}\left(\beta \right),\;\beta =k\sqrt{{\left(\xi -x\right)}^{2}+z^{2}},$$ (10a)

where $H_{0}^{\left(1\right)}=J_{0}+{\text{iY}}_{0}$ is the Hankel function of the first kind and zero order [19, 20], J_0_ and Y_0_ are the corresponding Bessel functions of the first and second kind, and *β* is the same as in Eq. (8a). Hence the solution for $u_{\text{RS}}^{\left(s\right)}$ is obtained at once by substitution of $\partial H_{0}^{\left(1\right)}/\partial z=-k^{2}zH_{1}^{\left(1\right)}/\beta$ into Eq. (10a), leading to

$$u_{\text{RS}}^{\left(s\right)}\left(x,z\right)=\frac{1}{ik}\frac{\partial u_{\text{RS}}^{\left(p\right)}}{\partial z}=\frac{ik^{2}z}{2}\int_{-w+x}^{w-x}d\left(\xi -x\right)\frac{H_{1}^{\left(1\right)}\left(\beta \right)}{\beta }.$$ (10b)

**Fig. 7:**
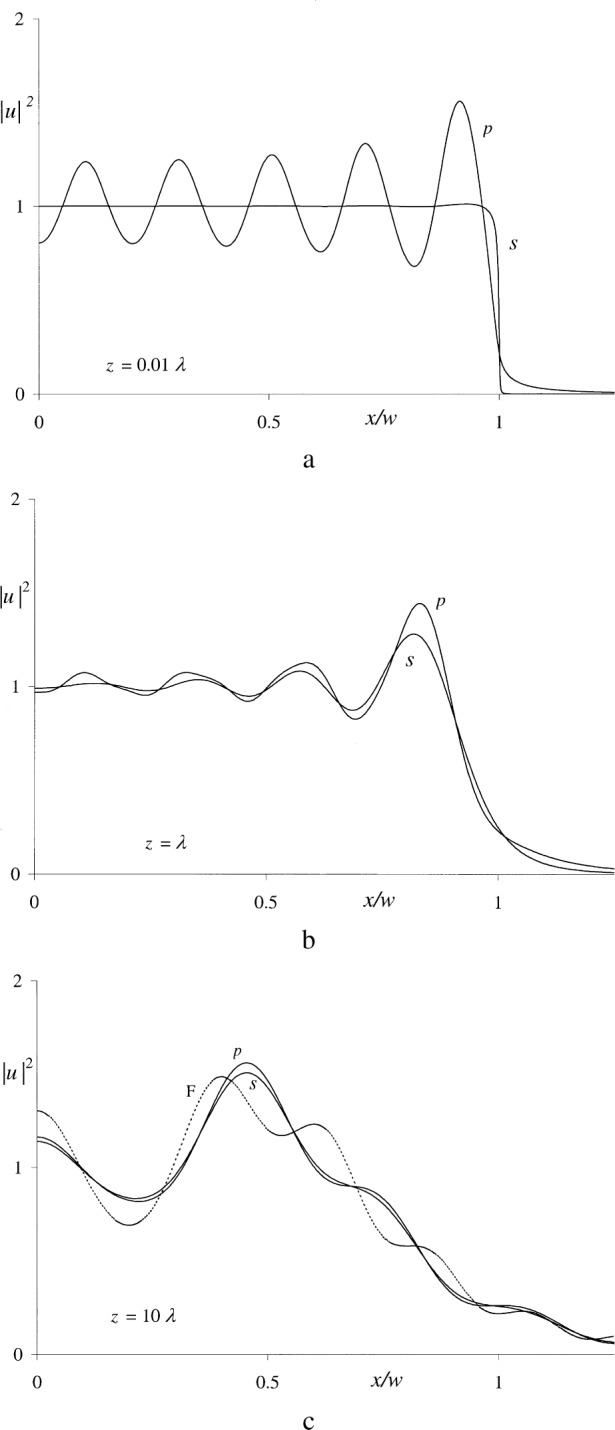
Diffraction profiles for a slit of width 2*w* = 10λ at distances *z* ranging from 0.01λ to 10λ. The profiles shown in each graph are the squared magnitudes of the Rayleigh-Sommerfeld solutions $u_{\text{RS}}^{\left(p\right)}$ and $u_{\text{RS}}^{\left(s\right)}$ (labeled *p* and *s*), and in addition the dotted line in Fig. 7c represents the Fresnel approximation *u*_F_ (labeled F).

These expressions were evaluated by numerical integration, again using the methods of Ref. [18] and assuming 2*w* = 10λ, *z* = 0.01λ, λ, and 10λ. The results obtained are shown in Figs. 7a through c. It should be noted that, in spite of the singularities of and $H_{0}^{\left(1\right)}\left(\beta \right)$ and $H_{1}^{\left(1\right)}\left(\beta \right)/\beta$ at *β*= 0, the computations for *z* = 0.01λ presented no problems as long as sufficiently small summation elements [Δ(ξ *− x*) = 0.01*w*] were used.

## 4. Discussion

The mathematical expressions derived in the previous Section proved their worth for practical applications, in that the computation of the diffraction profiles plotted in Figs. 5 and 7 posed no problems. The results obtained were everywhere finite, free from singularities, and provided new insight into the nature of diffraction in the close proximity of apertures. In spite of the obvious differences between the profiles pertaining to circular apertures slits, their over-all behavior in the near zone is similar so that it may be conjectured that the following observations are not restricted to these specific aperture forms.^2^

1. As was to be expected, $u_{\text{RS}}^{\left(s\right)}$ replicates the assumed rectangle functions Eqs. (6a, b) in the limit *z* → 0. However, it does not constitute an analytical continuation of the incident field into the half space *z* > 0 because otherwise $\partial u_{\text{RS}}^{\left(p\right)}/\partial z$, and thus $u_{\text{RS}}^{\left(p\right)}$, would also replicate their corresponding boundary values. On the other hand, the discontinuities of $u_{\text{RS}}^{\left(p\right)}$ do not manifest themselves in the form of sudden jumps, as might be surmised from the “*saltus*” interpretation of Kirchhoff’s theory. Instead, they are oscillatory in nature and reminiscent of the manner in which the rectangle functions Eqs. (6a, b) might be approximated by a Fourier series.
2. When *z* increases, $u_{\text{RS}}^{\left(p\right)}$ and $u_{\text{RS}}^{\left(s\right)}$ gradually converge to the Fresnel’s integral in Eq. (3). For the aperture width 10λ assumed in the examples the Fresnel limit is expected to be reached when *z ~* 100*λ*, and yet Figs. 5c and 7c show that the differences between $u_{\text{RS}}^{\left(p\right)}$ and $u_{\text{RS}}^{\left(s\right)}$ are already very small at only one tenth this distance. This suggests that, according to the Rayleigh-Sommerfeld theory, polarization effects are negligibly small even in the near zone.

Although the mathematical expressions derived in this paper will be useful for computations in the near zone, it remains unclear which of them ought to be used in given cases. In a pragmatic sense this may be a mute question, because $u_{\text{RS}}^{\left(p\right)}$ and $u_{\text{RS}}^{\left(s\right)}$ are so similar in most of the near zone that either will be an improvement over the Fresnel approximation and it does not matter which is used. Therefore, $u_{\text{RS}}^{\left(s\right)}$ could be (and has been) regarded as the preferred solution as it is continuous in the aperture plane, or Kirchhoff’s solution *u*_K_ could be regarded as a best compromise as it is the arithmetic mean of $u_{\text{RS}}^{\left(p\right)}$ and $u_{\text{RS}}^{\left(s\right)}$. In this author’s opinion, these are unfounded guesses. The fact of the matter is that assessing the physical significance of the Rayleigh-Sommerfeld integrals requires additional considerations that will be the subject of a subsequent paper.

## Footnotes

1 *Saltus* is Latin for *jump* or *leap*. This author prefers the maxim that *natura non facit saltus*.

2 Although Kirchhoff’s solution is not explicitly mentioned here, its behavior can easily be deduced as it is the arithmetic mean of the Rayleigh-Sommerfeld integrals.
